# Rethinking resilience and development: A coevolutionary perspective

**DOI:** 10.1007/s13280-020-01485-8

**Published:** 2021-02-10

**Authors:** L. Jamila Haider, Maja Schlüter, Carl Folke, Belinda Reyers

**Affiliations:** 1 Stockholm Resilience Centre, Stockholm University, Kräftriket 2B, 10691 Stockholm, Sweden; 2 The Beijer Institute of Ecological Economics, The Royal Swedish Academy of Sciences, 10405 Stockholm, Sweden; 3 Future Africa, University of Pretoria, Hillcrest Campus, Pretoria, South Africa

**Keywords:** Coevolution, Development, Filtering, Resilience capacities, Social–ecological

## Abstract

The interdependence of social and ecological processes is broadly acknowledged in the pursuit to enhance human wellbeing and prosperity for all. Yet, development interventions continue to prioritise economic development and short-term goals with little consideration of social-ecological interdependencies, ultimately undermining resilience and therefore efforts to deliver development outcomes. We propose and advance a coevolutionary perspective for rethinking development and its relationship to resilience. The perspective rests on three propositions: (1) social-ecological relationships coevolve through processes of variation, selection and retention, which are manifest in practices; (2) resilience is the capacity to filter practices (i.e. to influence what is selected and retained); and (3) development is a coevolutionary process shaping pathways of persistence, adaptation or transformation. Development interventions affect and are affected by social–ecological relationships and their coevolutionary dynamics, with consequences for resilience, often with perverse outcomes. A coevolutionary approach enables development interventions to better consider social–ecological interdependencies and dynamics. Adopting a coevolutionary perspective, which we illustrate with a case on agricultural biodiversity, encourages a radical rethinking of how resilience and development are conceptualised and practiced across global to local scales.

## Resilience capacities and development pathways

There is increasing recognition of the need for development (including sustainable and international development efforts) to better acknowledge and address the inextricability of social and ecological processes, from local to global scales. This recognition is critical in order to enhance human wellbeing without eroding the dynamic relationships between people and nature, upon which it depends (Reyers et al. 2018; Díaz et al. 2019).

This is especially relevant when considering complex development challenges such as food and water security, which are further confounded by emerging crises including novel pandemics, rising inequalities and climate change (Johns and Sthapit 2004; Nyström et al. 2019). The question thus arises, how should development practitioners engage to enhance human wellbeing without eroding critical social–ecological processes, and especially the dynamic relationships between them?

Resilience has been proposed as a way to address this question: to cultivate the capacity to maintain social–ecological relationships (Brown and Westaway 2011), to better integrate cultural, ecological and economic dynamics in development (Lade et al. 2017) and to deal with complexity and uncertainty (Ramalingam et al. 2008; Levin et al. 2013). Over time, resilience has become an increasingly popular term in development practice and research. While initially these efforts tended to focus narrowly on resilience as an outcome (Maxwell et al. 2011), more recent approaches have implemented resilience as capacities of an individual, a community or a system to persist, adapt or transform (Béné et al. 2014; Bousquet et al. 2016; Brown 2016; Folke et al. 2016; Jeans et al. 2016; Lade et al. 2020). The notion of resilience as capacity has long been a focus in ecosystem management and natural resource governance (Olsson et al. 2004), where resilience is defined as “the capacity to adapt or transform in the face of change in social–ecological systems, particularly unexpected change, in ways that continue to support human well-being” (Folke et al. 2016). Resilience, as we use it here, is the result of a combination of three capacities which lead to different responses: (1) absorptive capacity leading to persistence, (2) adaptive capacity which leads to incremental adjustments and adaptive changes, and (3) transformative capacity leading to structural or systemic reconfigurations.

There is a need for conceptual and operational tools to better understand and apply resilience as capacities to shape development options and pathways. Specifically, we need better understanding of the mechanisms through which pathways of persistence, adaptation or transformation emerge and are shaped (Few et al. 2017; Schlüter et al. 2019; Scoones et al. 2020). We propose that a coevolutionary perspective offers an avenue for understanding and enacting resilience capacities to shape development pathways.

Coevolution is a theory that addresses how different entities or relationships mutually influence each other’s evolution. Coevolutionary processes are inherently dynamic, and a coevolutionary perspective specifies the mechanisms that shape how properties, processes and innovations are maintained or varied over time. Here we demonstrate how coevolution is a useful conceptual tool for rethinking development as a dynamic process of coevolving social–ecological relationships. For example, agricultural practices (such as traditional practices of sowing a diversity of seeds, or sowing an improved variety of seed) can be understood as coevolving with the landscape, as the practice shapes the landscape and the landscape shapes the practice.

This perspective paper aims to bring together coevolution, development and resilience research to further understanding of the capacities to persist, adapt and transform, and their role in shaping development pathways. We present an overview of coevolution literature, followed by three propositions outlining a social–ecological coevolutionary approach. From this, we explore implications for development practice in an agricultural case study.

## A coevolutionary understanding of resilience

Coevolution is a process of open and non-deterministic change between culture, practices and biophysical environments that mutually influence each other’s evolution. Humans change environments, and in turn, environments change human practices and ideas (Kallis 2007; Schill et al. 2019). Key evolutionary mechanisms are variation, selection and retention (Kallis and Norgaard 2010). A recent resurgence of coevolutionary theory has demonstrated its applicability to studying dynamic social–ecological relationships in pursuit of sustainability challenges (Weisz et al. 2011; Søgaard Jørgensen et al. 2020). Table 1 draws on different traditions in order to bring together useful insights from coevolution for resilience and development.

**Table 1:** Overview of coevolution across different fields

Field	Definition	Key literature
Ecology	When members of two species interact, the change in each produces alterations in the life of the other, and each may generate selective forces that direct the evolution of the other	Ehrlich and Raven (1964), Begon et al. (2006)
Cultural	Micro agency-centred perspective; specifying the means of coevolution at individual level through ‘everyday practice’: “… a sensible theory of cultural evolution will have to explain why some beliefs and attitudes spread and persist and others disappear. The processes that cause such cultural change arise in the everyday lives of individuals as people acquire and use cultural information. Some values are more appealing and thus more likely to spread from one individual to another. These will tend to persist, while others disappear” (Richerson and Boyd 2005, pp. 6)	Richerson and Boyd (2005)
Biocultural	Diversity of life in all its manifestations—biological, cultural and linguistic—which are interrelated within a complex social–ecological adaptive system and that have coevolved with each other	Smith (2001), Maffi (2005)
Geography	Coevolution in geography has seldom expanded beyond the social sphere but increasingly sees social–ecological coevolution as a frontier. In a coevolutionary process autonomous but dialectically interwoven elements, moments or spheres of activity constitute a social–ecological totality of ensembles or assemblages	Weisz et al. (2011)
Socio–technical systems	Mainly focused on coevolution within social systems, e.g. between technology and society. Coevolutionary dynamics across multiple levels reinforce each other and enable transitions. Variation can also be created by active search for new solutions	Geels (2006, 2010)
Ecological economics	Humans change environments both materially and cognitively and in turn, new environments change human practices and ideas	Norgaard (1994), Kallis (2007), Kallis and Norgaard (2010)
Social–ecological	Emphasis on intertwinedness of social and ecological systems. The coevolutionary character of social–ecological systems reflects the fact that they can change qualitatively to generate novel outcomes.	Berkes et al. (2003), Waring et al. (2015)

Our application of coevolution theories to understand resilience and development builds on previous work (Table 1), drawing specifically on the conceptualisations of social–ecological systems and biocultural traditions of coevolution to highlight the interdependence of nature and culture (Maffi 2005). In addition, we find useful the perspective from geography of the rejection of ecological and cultural systems as separable entities, proposing instead a focus on processes and dynamic relationships that constitute a social–ecological whole (Weisz et al. 2011). The socio-technical systems definition of coevolution helps conceptualise it as a multi-level process, and how institutional structures and governance interact with technology (Geels 2006, 2010). Marrying such diverse conceptualisations, albeit from overlapping discursive communities, calls for reflexivity and methodological pluralism in research and practice (Norgaard 1994).

Coevolution is related to other concepts such as adaptive co-management, which involves a feedback between management policy by collaborating actors and the state and the dynamics of a resource (Olsson et al. 2004). Coevolution is also key to work on traditional ecological knowledge systems where practices, and the social mechanisms behind those practices, reflect a coevolutionary relationship between local institutions and the ecosystems in which they operate (Berkes et al. 2003). In contrast to coevolution, co-development is when there are direct effects between a societal action and environmental impact but not necessarily bidirectionally causal (Malerba 2006). While similar to these concepts, coevolution further adds notions of innovation, variation and indirect effects of selection pressures (for example, through processes of trial and error resulting in the selection of more sustainable practices over others (Colding and Folke 1997)).

## Development as coevolution: Three propositions

A coevolutionary perspective helps conceptualise development in a dynamic and social–ecologically interdependent way by explicitly invoking processes of variation, selection and retention. The three propositions below put forward how social–ecological systems coevolve, how resilience capacities act as a filter and how development interventions coevolve with development outcomes over time.

### Proposition 1: Social–ecological relationships coevolve through processes of variation, selection and retention, and are manifest in daily practices

**Fig. 1:**
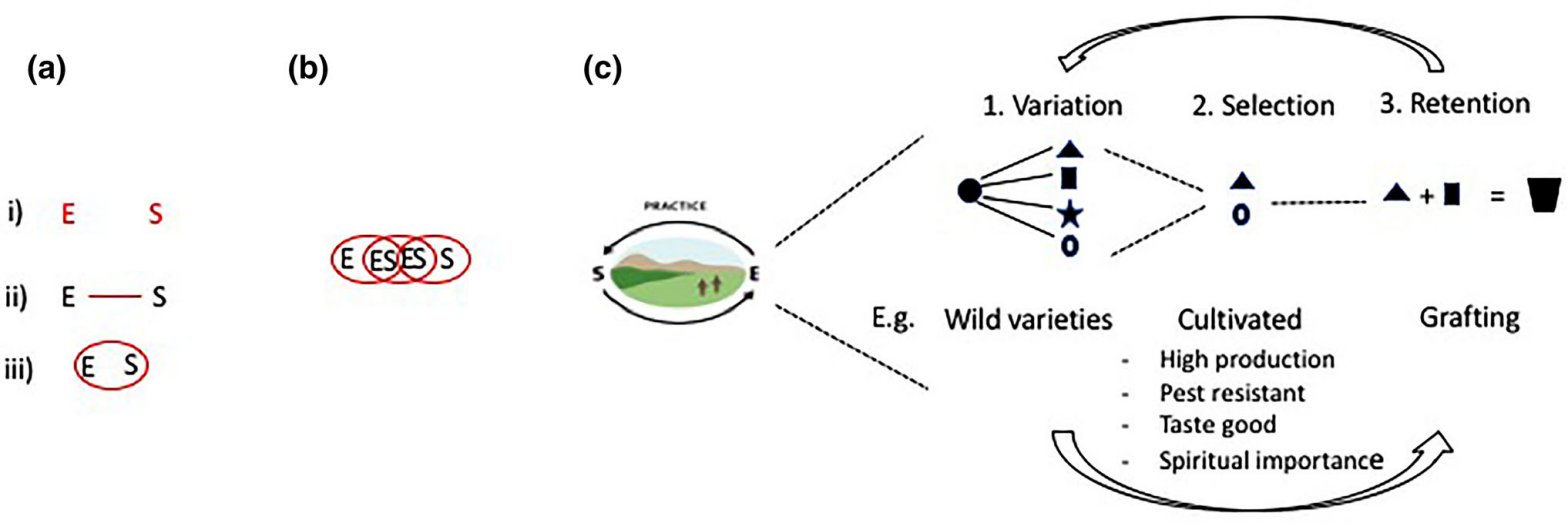
In the left panel (**a**) there are different representations of relations between ecological (E) and social (S) processes from (i) E and S as separate entities (in red), to (ii) linking (in red) E and S, and (iii) finally ES as inextricably intertwined (circle in red). In panel (**b**), a representation of social–ecological relationships as coevolved. **c** Practice can be considered to be a unit of coevolution, in which variation, selection and retention act and feedback on each other. We provide an example of the coevolution of a fruit tree in panel **c**

Instead of depicting the environmental and social features of a system as abstract entities that exist in isolation (Fig. 1a), a coevolutionary approach depicts the social and environmental in relation to one another and in constant interplay. Understanding these relationships as continuously coevolving helps redefine development processes as dynamically interdependent. Coevolution can occur between perceived ecological and social entities (for example, between crops and cultural practices (Fig. 1, panel a)), where researchers or practioners choose to focus their analysis on either (i) change in the entities themselves, (ii) in the relationship between them, or (iii) a social–ecological system as co-constituted, and in which (Fig. 1, panel b) coevolution occurs within relations (i.e. observable as practice). From Table 1, it is clear that there are ontological tensions among different perspectives of coevolution, particularly with respect to the existence of coevolving entities versus coevolving relations. These should be engaged with pragmatically. We suggest that these relations can be observed and interpreted through the concept of ‘practice’ - as in Norgaard (1994), Richerson and Boyd (2005) and as in Gidden’s concept of Practical Consciousness (1986).

We define practice as the social–ecological interaction that results in selection and retention during a specified event. Figure 1c provides an example of the coevolution of social–ecological practices. A diversity of wild plants (apple tree cultivars, for example) is the result of variation and is the raw material of an evolutionary process. Selection occurs through a variety of environmental and social factors, for example, drought resistance or preferred taste of a particular wild variety. From the selected apple varieties, one is retained and multiplied across the region. An innovative farmer may retain and graft a new marketable variety of apple onto the native root stock (note that retention is driven here by a social practice, not through genetic flows). The result of this fusion is a new variety of apple (Giuliani et al. 2011), starting the iterative process of variation.

### Proposition 2: Resilience is the capacity to filter practices

In line with recent advances, we propose that resilience can be usefully conceptualised as the capacity to adapt to change or transform by shaping and filtering practices (i.e. to influence what is selected and retained).

**Fig. 2:**
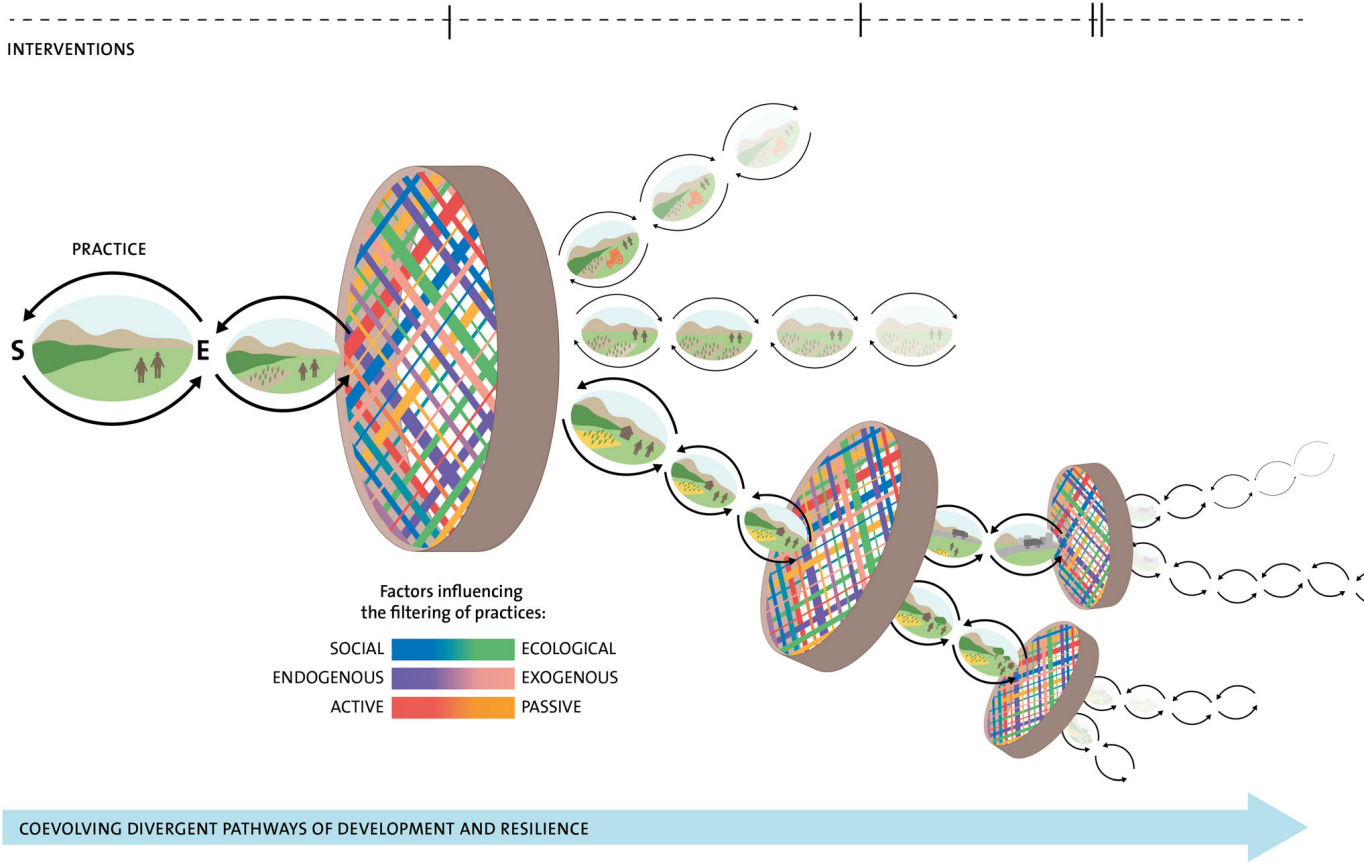
Coevolving pathways of development. The filters in the figure represent the selection and retention of practices which occur as the result of an external intervention, which lead to new variation in future development options or pathways. Practices are filtered by a set of constraints, opportunities and choices defined by myriad factors, ranging from social to ecological, endogenous to exogenous and active to passive (represented by different colours). Each filtering process creates the conditions for new development pathways to emerge. The filters are drawn as traditional winnowing baskets as used in the Pamirs Mountains (Figure redrawn from (Haider 2017))

Resilience in this sense is not inherently normative but becomes so depending on how capacity *of what* to adapt or transform *to what* is normatively defined. We argue that a coevolutionary perspective reframes resilience away from an outcome, to a complex capacity which influences development pathways. Filtering determines which components of existing practices are retained or discarded and which new components of practice are selected (Fig. 2). The filter represents the resilience capacities that are present and active at a particular moment in time. We choose ‘intervention’ here as a specific moment in time (event) which prompts the explicit representation of a filter, whereas in reality, the filters are constantly recurring and coevolving in an ever unfolding process. The filtering process is influenced by a number of factors: social–ecological relationships, power which can be situated more endogenously or exogenously, and both active and passive drivers. Differences in influence can be linked to biophysical constraints, or can be linked to differing power dynamics among actors, where the ideas of powerful development agencies may often have priority over ideas buried in tacit knowledge of local stakeholders. For example, some farmers may choose to actively keep ‘old’ customary practices, or select and retain new practices that are perceived as beneficial, thereby mixing old and new, endogenous and exogenous elements in order to create novel pathways (akin to *bricolage* (Cleaver 2012)). Not only social factors influence the filtering process but also linked ecological processes as well (Mancilla Garcia et al. 2019). The lens of practice enables these intertwined processes to be mutually considered (Berkes et al. 2003, Darnhofer 2020). While we propose that a coevolutionary perspective provides a powerful lens to conceptualise social–ecological relationships in more dynamic ways, we are not here advocating evolutionary explanations of human social behaviour. When unravelling the different coevolutionary processes that lead to change in practices or lack, thereof, it is important to draw on different social and natural science theories, including theories on agency, power, knowledge and human behaviour (as they are, for example, in Cooke et al. (2016) and Schill et al. (2019).

Understanding changes to development and resilience as coevolving processes that are filtered in this way could help inform development practice through making explicit the process by which resilience capacities influence development, as well as the processes by which these capacities are built or eroded. These are clarified through two main contributions: (1) by raising awareness of the dynamic and relational nature of development as shaped by filters emphasising the need to better understand what the elements and processes of the filters are at particular moments in time, and (2) to understand how resilience capacities as filters of development pathways are built and eroded. These contributions better account for various direct and indirect processes that may affect a development pathway in response to exogenous interventions or endogenous change and can particularly help to draw out the practices and associated knowledge that normally hold less power.

### Proposition 3: Development is a coevolutionary process shaping pathways of persistence, adaptation or transformation

Instead of depicting development as progress towards an outcome (with resilience as a way to buffer that progress from turbulence), coevolution depicts how resilience capacities filter and therefore direct development pathways (Fig. 2). The capacities evolve over time via dynamic, deliberative and non-deliberative processes. Novel practices that are the outcomes of interventions coevolve with existing practices and thereby change the starting point for future interventions. Viewing development as a dynamic process of coevolution (of practices) mediated by resilience capacities, shifts the focus from only understanding what constitutes a desired outcome, to how resilience and development emerge.

Perceiving change as a coevolutionary process helps us understand how different development pathways emerge, some which may be undesirable and maintain the status quo such as a poverty trap, others transformative and reconfigure the social–ecological system. In contrast to a reductionist understanding of development, where the ‘parts’ of the system that are targets of change (e.g. beneficiaries of poverty alleviation) are not linked to the broader systemic context, a coevolutionary perspective requires explicit acknowledgement of dynamic and complex relationships, as well as the inherently unpredictable nature of development pathways.

The pathways in Fig. 2 depict how practices change over time, with increased field size for example. Following the first filter in Fig. 2 (top pathway), a tractor is introduced but eventually that pathway fades away, perhaps because tractors are not well suited to the steep slopes of the fields in this context and contribute to soil erosion. This leads to path dependence, where diverse opportunities have in the meanwhile been diminished, for example, loss of diverse local agroecological practices (Lade et al. 2020). In the lower development pathway (Fig 2), a new crop is introduced and some houses are built as a consequences of new livelihood opportunities. The pathway further diverges through a second filter, with the upper pathway showing the impact of a new road, and the lower pathways depicting an agroecological landscape, with the introduction of some trees. The process of filtering mediates a constant interplay between reducing and expanding options.

### Three propositions applied to an agricultural example

The importance of crop diversity, especially in the context of a changing climate with warmer temperatures and prolonged intense droughts, is broadly recognised (Lin 2011). A diversity of crops can increase the resilience capacity to respond to a variety of different stresses or shocks (Meldrum et al. 2017). Crops eaten all around the world today are the product of thousands of years of variation, selection and retention. While food calories, protein, fat and weight are increasing, global crop diversity is rapidly homogenising (Khoury et al. 2014). This homogenisation is due to agricultural expansion and intensification to produce more food, even though evidence suggests that food scarcity issues are often rather products of inequity and distribution (Garnett 2013). It remains unclear how global and regional climate change will affect agricultural production around the world and which crop varieties will emerge as important to meet future food security demands. In response, ex-situ seed conservation initiatives have been set up (e.g. the Svalbard Global Seed Vault) to safeguard crop diversity. However, the knowledge and culture which coevolved with varietal use of crops and the ecological conditions within which they grow, cannot be stored in seed banks but must be regularly practiced. Biocultural landscapes and similar agroecological approaches are, therefore, critically important, not only for the maintenance of global crop diversity but also for the cultural diversity necessary to adapt to a changing climate (Brondizio and Le Tourneau 2016). Drawing from research from the Pamir Mountains, a centre of origin of various global staple crops and a centre of high biocultural diversity (Vavilov 1917; Nabhan 2009) and also the poorest area of Central Asia subject to many development interventions (Middleton 2016; Haider et al. 2019), we explore each proposition in turn.

#### Proposition 1: Social–ecological relationships coevolve

Figure 1 provides an example of social–ecological coevolution in a biocultural landscape. In the Pamir Mountains, over 33 commonly cultivated apple varieties have emerged through social–ecological coevolution (Giuliani et al. 2011). From a coevolutionary perspective, the evolutionary forces are not only relevant to the variation, selection and retention of epigenetic material, but it is also the cultural practice of cultivating a particular apple variety that is retained. Daily farming practices, such as growing, harvesting and preparing food (Fig. 2a), are defining features of landscapes and offer tangible manifestations of social–ecological relationships that coevolve. For example, the loss of a single seed variety may not seem like a major loss, but through observing practice, the interrelatedness of that seed to culture, spirituality and social organisation becomes apparent. The wealth of knowledge in how to care for, prepare and celebrate that seed emerges from the practices of farmers in their landscapes and in their cultural rituals (Haider et al. 2019). A development intervention that sees ‘seed’ solely as a productive input (and not as an embodiment of all the relationships that have created and maintained that seed) risks eroding biocultural diversity (Wiggins and Cromwell 1995; Fischer and Hajdu 2015; Lade et al. 2017; Haider et al. 2019). A shift from treating seed merely as an input, to seed as part of broader social–ecological relationships can be achieved by observing and actively participating in practice.

#### Proposition 2: Resilience is the capacity to filter practices

Development interventions taking a coevolutionary lens would support the resilience capacities that maintain practices characterised not only by efficiency but also diversity and redundancy. For example, a development intervention of an improved seed (exogenous factor) aiming to alleviate poverty through increased productivity, introduces a new practice. This practice will be filtered by existing resilience capacities and in turn can potentially change these capacities, which would lead to a profound influence on farming practices, resilience and development outcomes. Often, the use and spread of the improved seed are supported by powerful actors which influence the filtering process in favour of the improved seed over local practices and varieties. The improved seed thus has a higher chance of retention than those seeds borne from local and traditional knowledge. A woman in a community growing her own landrace seeds (locally adapted and domesticated), for instance, may not have the same influence that a development organisation has to mould the filter so that her ideas and practices can survive. Her practices may not be selected or retained, and thereby the innovative potential for variation also disappears.

#### Proposition 3: Coevolutionary development pathways of persistence, adaptation or transformation

The sowing, harvesting, storing and preparation of food are examples of social–ecological practices that have coevolved with landscapes over millennia in response to changing environmental and social contexts and needs, and are thus a source of memory and innovation (Berkes et al. 2003). The social–ecological memory embodied in these practices enables a diversity of responses to endogenous or exogenous change, generating variation. This ability to hold memory and renewal in tension is a core tenet of resilience in that historic knowledge and practices provide the seeds of innovation for the future (Gunderson and Holling 2002). In a coevolutionary process, selection, retention and variation act on each other to create constant change, characterised by persistence, adaptation or transformation (proposition 3). Haider et al. (2019) show how an intervention of an improved wheat seed variety 20 years ago in the Pamirs introduced to two different communities underwent different filtering processes in each. This resulted in two distinct development pathways: one of persistent food insecurity and one of adaptation. The first community lost their traditional seed varieties after trying the improved variety (which failed after two years) and now rely on food aid imports, while maintaining traditional practices and rituals disconnected from the ecological reality. The second community maintained their traditional varieties and practices and had local wheat varieties to fall back on when the improved seeds failed. This demonstrates how development pathways are coevolutionary, as improved seed and ideas coevolved with values and governance systems.

## Conclusions and Implications for development practice

Development is often reduced to a set of simple targets linked to interventions to improve wellbeing, alleviate poverty or address inequity. Narrow development interventions risk not only being ineffective but may also lead to unintended consequences. We suggest that development can be fundamentally redefined as a coevolutionary process of social and ecological interdependence, in which interventions shape outcomes, which in turn shape future development pathways.

Phenomena such as persistent poverty and chronic food insecurity are frequently blamed on factors such as ‘poor initial conditions’ (Lybbert et al. 2004; Naschold 2012), and there are few attempts to understand the causal mechanisms that link those initial conditions to outcomes, thus impeding more systemic and ultimately effective interventions (Haider et al. 2018). As opposed to assuming development outcomes as pre-determined based on initial conditions, a coevolutionary approach encourages a complex adaptive systems perspective which emphasises the role of emergence, non-linearity, adaptation and cross-scale interactions in determining how future outcomes interact with practices (Levin et al. 2013) and their implications for sustainable development (Reyers et al. 2018). Attempts to bridge the ecological and social domains towards a more systemic perspective in sustainable development through Agenda 2030 have led to a focus on interlinked “people, planet and prosperity”. Its enactment, however, reverts back to a focus on 17 goals and 169 targets, which largely separate ecological and social processes, and ignore cultural dynamics (Poole 2018; Reyers and Selig 2020), and therefore risks failing to consider key interdependencies in how development pathways actually unfold. A coevolutionary perspective redefines development as a constantly changing process but goes beyond that to explicitly recognise the influence of social–ecological dynamics which can be navigated by resilience capacities, thereby helping understand and design diverse development pathways of persistence, adaptation or transformation.

A coevolutionary perspective to development uses existing social–ecological relationships or practices as entry points, acknowledging that old and new practices will coevolve with each other to create entirely novel pathways, and considers the myriad factors which shape resilience capacities and ultimately influence which practices are selected and retained. Development interventions adopting a coevolutionary approach can thus more effectively enhance and support the dynamic social–ecological relationships that underpin wellbeing, shape resilience capacities and increase the opportunity space for development.
